# Clinical and microbiological evaluation of non-surgical periodontal therapy in obese and non-obese individuals with periodontitis: a 9-month prospective longitudinal study

**DOI:** 10.1590/1678-7757-2019-0694

**Published:** 2020-05-18

**Authors:** Felipe da Silva PERALTA, Sheila Cavalca CORTELLI, Emanuel Silva ROVAI, Davi Romeiro AQUINO, Taís Browne MIRANDA, Fernando Oliveira COSTA, José Roberto CORTELLI

**Affiliations:** 1 Universidade de Taubaté Departamento de Odontologia TaubatéSão Paulo Brasil Universidade de Taubaté , Departamento de Odontologia , Taubaté , São Paulo , Brasil .; 2 Universidade Federal de Minas Gerais Faculdade de Odontologia Departamento de Periodontia Belo HorizonteMinhas Gerais Brasil Universidade Federal de Minas Gerais , Faculdade de Odontologia , Departamento de Periodontia , Belo Horizonte , Minhas Gerais , Brasil .

**Keywords:** Obesity, Periodontitis, Body mass index, Periodontal disease

## Abstract

**Objective:**

Obesity is a chronic disease that negatively affects an individual’s general and oral health. The present study aimed to compare the clinical and microbiological effects of non-surgical periodontal therapy with the full mouth disinfection (FMD) protocol on obese and non-obese individuals at 9 months post-therapy.

**Methodology:**

This clinical study was first submitted and approved by the Ethics Committee. Fifty-five obese patients and 39 non-obese patients with periodontitis were evaluated. The full-mouth periodontal clinical parameters, clinical attachment level (CAL), probing depth (PD), gingival index (GI), and plaque index (PI), were monitored at baseline, 3, 6, and 9 months after periodontal treatment with full mouth disinfection (FMD) protocol. The mean count of *Tannerella forsythia* , *Porphyromonas gingivalis* , *Treponema Denticola* , and *Aggregatibacter actinomycetemcomitans* was determined by quantitative polymerase chain reaction on subgingival biofilm samples. Demographic data were assessed by Chi-square test. For clinical and microbiological parameters, two-factor repeated-measures ANOVA was used.

**Results:**

In both groups, periodontal therapy using the one-stage full-mouth disinfection protocol significantly improved CAL, PD, GI, and PI (p<0.05). Obese and non-obese patients equally responded to non-surgical periodontal therapy (p>0.05). Microbial count found no major differences (p>0.05) between obese and non-obese individuals who had undergone non-surgical periodontal therapy.

**Conclusions:**

Obesity did not affect the clinical and microbiological outcomes of non-surgical periodontal therapy.

## Introduction

Periodontitis is a highly prevalent chronic inflammatory disease characterized by the progressive destruction of the tooth-supporting tissues, resulting in tooth loss. ^1^ ^,^ ^2^ Periodontitis represents not only a simple bacterial infection, but also a complex interaction among host inflammatory responses, subgingival biofilm, and host modifying factors. ^3^

Among host modifying factors, several systemic diseases have been associated with periodontitis, including obesity. ^4^ ^,^ ^5^ Obesity is a chronic disease that affects the individual’s general and oral health. ^6^ ^,^ ^7^ The cellular and molecular mechanisms that might explain obesity and periodontitis relationship are systemic inflammatory changes in tumor necrosis factor (TNF)-α, Interleukin-6 (IL-6), oxidative stress, and adiponectin and leptin levels, which may result in greater susceptibility to chronic inflammatory diseases and infections. ^8^ ^-^ ^10^

Several epidemiological studies have established the relationship between obesity and periodontal disease. ^11^ ^-^ ^13^ Cross-sectional studies have demonstrated that obese individuals present greater odds-ratio for periodontitis than non-obese. ^11^ ^,^ ^13^ Moreover, longitudinal studies demonstrated that obesity is associated with increased periodontal attachment loss and inflammation, ^12^ ^,^ ^14^ ^,^ ^15^ and systematic reviews showed that periodontitis severity and extension are associated with increased levels of overweight. ^16^ ^-^ ^18^

Previous reports showed that obese individuals present impaired wound healing and more infectious complications. ^19^ ^,^ ^20^ Therefore, the question of whether these individuals would have a worse response to periodontal therapy than non-obese individuals was posed, and a few clinical studies were conducted. ^20^ ^-^ ^25^ Nonetheless, a systematic review evaluating obesity on periodontal therapy showed that the low quality of evidence is mainly due to the lack of studies, short-term follow-ups, and inconsistent data, indicating the need for further studies. ^26^ ^,^ ^27^

As inflammatory changes in obese individuals may impair wound healing and worsen periodontal status, this study hypothesized that obese individuals would present a worse response to periodontal therapy than non-obese ones. Although there are other studies on this topic, this is the first to verify the effectiveness of the chemical-mechanical therapy with the FMD protocol in obese individuals.

Thus, the aim of this prospective 9-month clinical study was to compare the clinical and microbiological effects of non-surgical periodontal therapy with the FMD protocol on obese and non-obese individuals.

## Methodology

This 9-month, parallel group, single-center, clinical study was registered at Clinicaltrials.gov (NCT03103204) and approved by the Ethics Committee (protocol No. 36828114.4.0000.5501). Participants provided prior written informed consent for enrolling in this clinical study, composed of baseline, 3, 6 and 9 month post-therapy follow ups.

### Participants

The study population was composed of participants with periodontitis referred to the Dental Specialties Center of Joinville-SC, in 2017. A total of 94 individuals of both genders, 45 years old or older, and presenting at least 12 natural teeth, body mass index (BMI) > 18.5 kg/m^2^ and periodontitis were recruited. This prospective study included participants with moderate, severe, and advanced periodontitis (stage II: established periodontitis with characteristic damages caused to tooth support, including interdental CAL from 3 to 4 mm, maximum PPD ≤ 5 mm, and radiographic bone loss at coronal third between 15% to 33%; stage III and IV: – at least interdental CAL ≥ 5mm, PPD ≥ 6 mm and radiographic bone loss extending to mild-third of the root), as described by Tonneti, et al. ^28^ (2018).

Sample size was calculated from the primary outcome data of previous studies, based on the clinical attachment level (CAL) gain and probing depth (PD) reduction. ^29^ ^,^ ^30^ Microbial count reduction and the association between clinical and microbiological reductions were chosen as secondary outcome, based on the *posteriori* mean microbial counts observed in this study. Assuming a 5% α, a 90% power, and a 15% minimum PD reduction difference between groups, a total of 28 individuals within each group would be necessary. Notably, in this study the coefficient of variation for microbial count was ~15%, indicating the study outcome precision. ^29^ Considering a dropout of up to ~20%, a total of 34 participants in each group were initially regarded as appropriate.

Exclusion criteria were orthodontic devices, pregnancy or breast-feeding, systemic diseases or other conditions that could influence the periodontal status (other than diabetes), alcohol abuse, medical condition requiring prophylactic antibiotic coverage, use of systemic antibiotics and/or anti-inflammatory drugs six months prior to the study, and history of periodontal therapy within six months prior to the study.

### Anthropometric measurements

Anthropometric measurements (weight, height, and waist circumference) were recorded at both baseline and 9 months. BMI was calculated as an indicator of total adiposity regarding obesity, by dividing bodyweight (kilograms) by the square of body height (meters). Obesity was defined as BMI ≥ 30 kg/m ^2^ . ^31^ Alongside BMI, the following waist circumference reference-values were considered as indicators of obesity: > 102 cm for men and > 88 cm for women. ^31^

Individuals selected were divided into two groups, according to their body mass index (BMI) and waist circumference.

Non-obese group (n= 39), BMI ≤ 29.9 kg/m ^2^ and waist circumference < 102 cm for men and < 88 cm for women.

Obese group (n=55), BMI ≥ 30 kg/m ^2^ and waist circumference > 102 cm for men and > 88 cm for women.

### Clinical procedures

Full mouth periodontal clinical parameters were obtained by a single previously trained examiner (F.S.P.), and subsequently calibrated by a gold-standard examiner (J.R.C.). The training and calibration processes followed the method described by Araujo, et al. ^32^ (2003) and were performed both prior to the study and before the final exam. An intra-examiner 0.85 correlation coefficient (Kappa test) for probing pocket depth measurements showed high examiner reliability.

Periodontal clinical parameters were obtained prior to therapy (baseline) and at intervals of 3, 6, and 9 months post-therapy. CAL, PD, plaque Index ^33^ (PI), and gingival index ^34^ (GI) were obtained with a manual periodontal probe (Hu-friedy – Chicago, IL, USA) of all teeth from six periodontal sites.

Initially, participants underwent oral hygiene instructions. Then, as described by Quirynen, et al. ^35^ (1995) _,_ the one-stage full-mouth disinfection protocol for periodontal therapy was performed by an experienced and trained periodontist (F.S.P). The protocol consisted of full-mouth periodontal debridement within 24 hours, in two sessions of one hour each, tongue brushing with 1% chlorhexidine gel for 1 minute, subgingival irrigation with 1% chlorhexidine gel after scaling, and mouthwashes with 0.12% chlorhexidine for 30 seconds at the beginning and at the end of each session, with gargling in the final 10 seconds. In addition, during fourteen days, twice-daily, 0.12% chlorhexidine was used. Every three months, patients underwent oral hygiene instructions, dental prophylaxis and supragingival dental scaling.

### Microbiological monitoring

As described in our previous study, subgingival plaque samples were collected. ^36^ Supragingival dental plaque was removed using a sterile curette, and a sterile paper point (fine, Johnson and Johnson, New Brunswick, NJ, USA) was inserted into the gingival sulcus/periodontal pocket and kept there for 10 seconds. Then, paper point was placed in a vial containing 1.0 ml of phosphate-buffered saline (pH7.4), inserted in a minitube and kept on ice. To obtain bacterial dispersion, a vortex mixer at maximum speed was used for 1 min and stored in a freezer at -80°C until further analyses.

Following the manufacturer’s specifications, the genomic DNA (gDNA) was extracted and purified using a commercial Genomic DNA Mini Kit (Life Technologies, Carlsbad, CA, USA).

**Figure 1:**
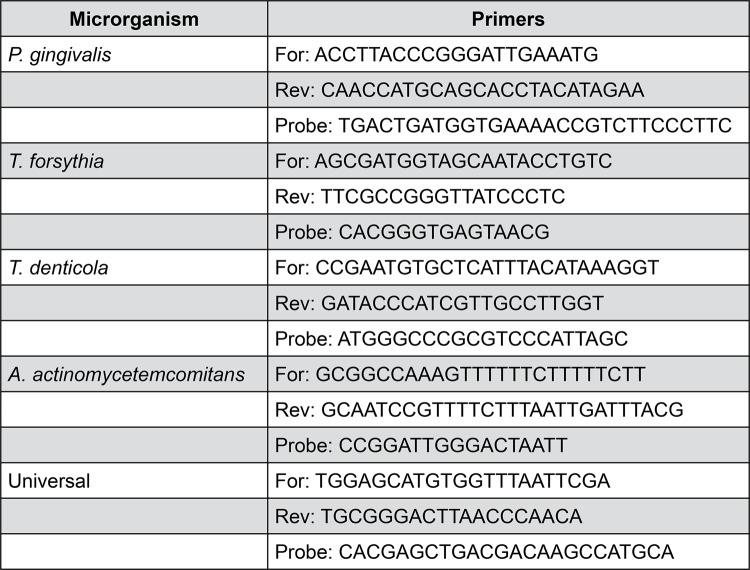
Specific primers and probes used in the study

The total microbial count of *Tannerella forsythia, Porphyromonas gingivalis, Treponema denticola* , and *Aggregatibacter actinomycetemcomitans* was performed by quantitative real-time polymerase chain reaction (qPCR) using a set of TaqMan (Life Technology, Carlsbad, CA, USA ) primers/probes in Real-Time PCR System, following manufacturer’s instructions. The qPCR conditions were: 50 ^o^ C for 2 min, 95 ^o^ C for 10 min, 40 cycles of 95 ^o^ C for 15 s, and 60 ^o^ C for 1 min. Figure 1 lists the primers and probes used in the study.

### Statistical analysis

Statistical analysis was performed by the GraphPad Prism statistical program (GraphPad Software, La Jolla, CA, USA). First, data were submitted to the Kolmorogov-Smirnov normality test. Demographic data were accessed by chi-square test to verify the group homogeneity. Patient served as unit to evaluate clinical parameters. Intra and inter-group comparisons were assessed by the two-factor repeated-measures ANOVA. Microbial counts were log-transformed, and a Q-Q plot of residual values showed acceptable levels of normality. All analyses were performed with 5% α.

## Results

**Figure 2:**
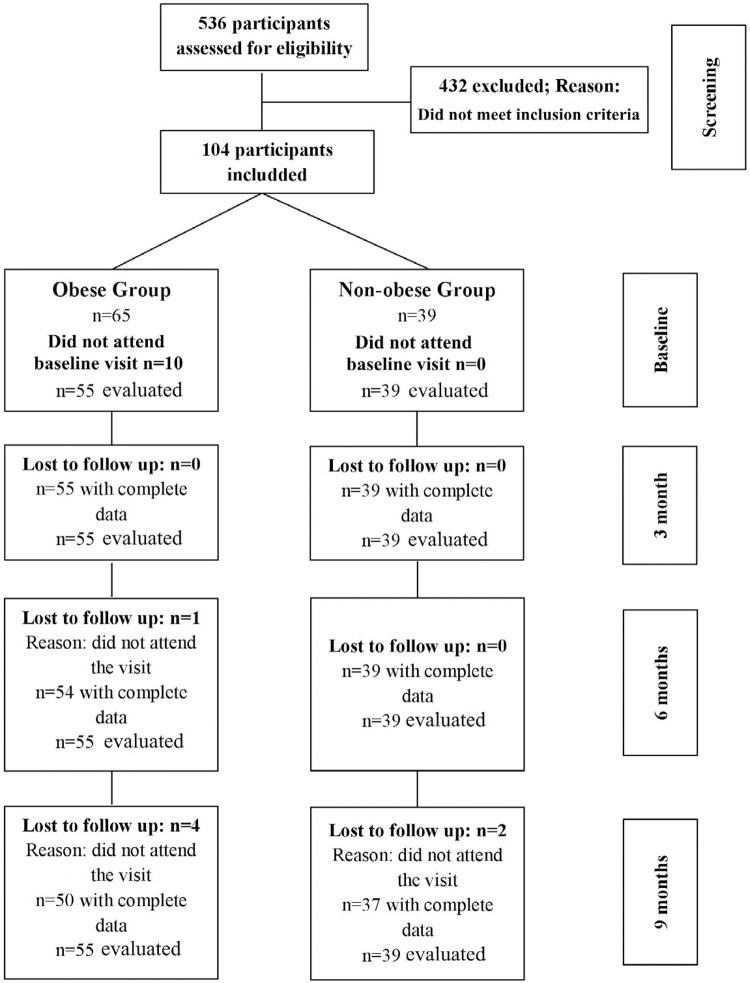
Flow chart of the study design

For this study, one hundred and four individuals were selected and divided into two groups (obese and non-obese). As ten subjects from the obese group did not attend the baseline appointment, only 94 participants were evaluated. Five individuals of the obese group and two of the non-obese were lost during follow-up visits. Figure 2 shows the flow diagram of the study design.

Table 1 shows participants’ sociodemographic and behavioral characteristics, and health conditions for both groups. There was no significant difference between the groups regarding age, sex, ethnicity, diabetes, and smoking habits. Obese individuals showed increased BMI and waist circumference. Moreover, the obese groups had a greater prevalence of participants with arterial hypertension (P=0.001).

**Table 1:** Sociodemographic, behavioral and health condition characteristics of Obese and non-Obese groups

Variables	Non-Obese (n=39)	Obese (n=55)	p-Value
Mean age in years (SD)	50.7(7.1)	48.9(7.8)	0.94
Gender: Male (%)	14(35.9)	19(34.5)	0.89
Ethnicity: Non-Caucasian (%)	3(7.7)	7(12.7)	0.51
BMI (mean SE)	25.8(0.5)	36.12(0.57)	0.001*
Waist circumference (mean SE)	90.34(1.86)	110.89(1.4)	0.001*
n (%) diabetes	2(5.1)	9(16.4)	0.11
n (%) of arterial hypertension	3(7.7)	30(54.5)	0.001*
Number (%) of smokers	5(12.8)	4(7.3)	0.48

*: Indicate significant differences between groups (p < 0.05).

n: number; SE: standard error; SD: standard deviation

All subjects reported no side effect caused by the treatment.

### Clinical outcomes

Table 2 shows periodontal clinical parameters observed in both groups at baseline, 3, 6, and 9 months post-treatment. Periodontal chemical-mechanical therapy using the one-stage full-mouth disinfection protocol significantly improved all clinical parameters (PD, CAL, GI, and PI) for both groups (p<0.05). However, the two of them equally responded to non-surgical periodontal therapy with no statistically significant differences in CAL, PD, GI, and PI after 9 months.

**Table 2:** Means (±SD) of full-mouth clinical parameters for both groups at baseline and follow-up visits

Variable	Time point	Non-Obese n=39	Obese n=55	p-values
PD(mm)	Baseline	2.98 ± 0.5	2.90 ± 0.3	0.553
	3 months	2.34 ± 0.4*	2.26 ± 0.3*	0.413
	6 months	2.22 ± 0.5*	2.24 ± 0.4*	0.87
	9 months	2.27 ± 0.5*	2.22 ± 0.4*	0.97
CAL(mm)	Baseline	4.23 ± 1.2	4.03 ± 0.9	0.525
	3 months	3.66 ± 1.2*	3.61 ± 1.0*	0.814
	6 months	3.64 ± 1.1*	3.50 ± 1.0*	0.549
	9 months	3.67 ± 1.1*	3.53 ± 1.0*	0.705
PI	Baseline	0.54 ± 0.32	0.51 ± 0.36	0.624
	3 months	0.45 ± 0.31*	0.39 ± 0.22*	0.25
	6 months	0.33 ± 0.23*	0.37 ± 0.21*	0.282
	9 months	0.32 ± 0.20*	0.32 ± 0.22*	0.813
GI	Baseline	0.32 ± 0.32	0.37 ± 0.25	0.431
	3 months	0.29 ± 0.19	0.28 ± 0.14*	0.662
	6 months	0.25 ± 0.20*	0.24 ± 0.26*	0.806
	9 months	0.27 ± 0.15*	0.23 ± 0.22*	0.218

*: indicate significant differences when compared with baseline by repeated measures ANOVA and Tukey’s tests (p < 0.05).

PD, probing depth; CAL, clinical attachment level; PI: plaque index; GI: gingival index; n: number; SD: standard deviation.

### Microbiological results

Table 3 shows microbial count at baseline, 3, 6, and 9 months. Total bacterial load was not significantly different among follow-up periods and groups (p<0.05). Within 9 months, *P. gingivalis* and *A. actinomycetemcomitans* significantly decreased in both groups (p<0.05) with no significant difference between groups. At 6 months, *T. forsythia* decresead significantly in the non-obese group, whereas for the obese group small counts were observed only at 3 months. These reductions, however, were not maintained in both groups at 9 months. Although no difference between groups was observed at any period, periodontal treatment reduced *T. denticola* count within the obese group ( *p* <0.05).

**Table 3:** Mean number of periodontal pathogens (SE) for both groups at baseline and at follow-up visits

Bacterial specie	Time point	Non-Obese n=39	Obese n=55	ANOVA p-values
*P. gingivalis*	Baseline	21.03(8.77)	17.06(4.62)	0.653
	3 months	17.07(10.53)	3.65(1.22)*	0.030#
	6 months	5.10(2.15)*	9.81(2.81)*	0.185
	9 months	10.58(4.79)*	13.88(5.49)*	0.72
*T. forsythia*	Baseline	50.38(21.51)	58.52(18.77)	0.785
	3 months	33.31(11.85)	22.05(7.20)*	0.376
	6 months	21.36(6.89)*	55.87(16.24)	0.025#
	9 months	45.70(21.50)	33.24(8.79)	0.533
*T. denticola*	Baseline	11.66(4.55)	18.14(6.01)	0.393
	3 months	6.52(2.22)	5.64(1.51)*	0.715
	6 months	9.60(2.80)	14.77(4.09)	0.279
	9 months	9.99(2.53)	7.14(2.32)*	0.482
*A. actinomycetemcomitans*	Baseline	73.36(11.75)	92.04(20.66)	0.227
	3 months	71.57(22.95)	65.55(36.59)	0.877
	6 months	46.85(18.09)*	48.22(15.36)*	0.954
	9 months	22.28(9.99)*	21.05(6.55)*	0.873
Total load	Baseline	11.85(2.41)	12.60(2.41)	0.818
	3 months	10.24(1.79)	7.75(1.33)	0.235
	6 months	9.12(1.70)	11.22(1.75)	0.39
	9 months	8.52(1.44)	9.10(1.61)	0.902

*: indicate significant differences over time by repeated measures ANOVA and Tukey’s tests (p < 0.05).

# : Indicate differences between treatment groups at each time point by repeated measures ANOVA and Tukey’s tests (p < 0.05).

n: number; SE: standard error

## Discussion

The objective of this 9-month clinical study was to determine whether obese individuals present a worse response to periodontal therapy than non-obese ones. Although few studies have been performed on this matter, this study was conducted because (1) the literature presents controversial data, ^26^ (2) most studies have 2-6 months follow-ups, ^22^ ^-^ ^25^ (3) studies combining mechanical and chemical procedures are lacking and (4) systematic reviews indicate the need for further studies. ^26^ ^,^ ^27^

The main findings presented here indicate that obesity (BMI > 30 kg/m ^2^ ) does not affect the clinical and microbiological outcomes of non-surgical periodontal therapy. As obesity has recently become pandemic, ^37^ groups were divided into non-obese and obese individuals to achieve a greater reproducibility and reliability for the overall population. Besides, a stratified preliminary analysis showed no significant difference between overweight and normal-weight for all parameters. Thus, as the aim was to assess whether obese people had a worse response to periodontal therapy than non-obese, overweight was included in the non-obese group. Although one may speculate that BMI is a general indicator of body size and not obesity, demographic data demonstrated significant differences in waist circumference between groups, considered a reliable indicator of intra-abdominal adipose tissue and increased risk of cardiometabolic disease. ^38^

Periodontal therapy using the one-stage full-mouth disinfection protocol effectively reduced periodontal inflammation, improving all clinical parameters (PD, CAL, and GI). Yet, obese and non-obese individuals showed no significant difference during the study follow-up period. These results might be explained by the disease low severity at baseline, as well as the presence of more smokers within the non-obese group, although not statistically different,. Nevertheless, these findings corroborate other clinical studies ^21^ ^,^ ^22^ ^,^ ^39^ and two systematic reviews, ^26^ ^,^ ^27^ which found that obese and non-obese subjects have a similar periodontal response to nonsurgical mechanical periodontal therapy. Conversely, other studies observed an impaired response to periodontal therapy in obese individuals. ^23^ ^-^ ^25^ These divergent results could be explained by: different follow-up periods, as some studies present 2 months, ^23^ 3 months ^21^ ^,^ ^39^ and 6 months follow-ups, ^24^ ^,^ ^25^ wheread this study had a 9-month follow-up; differences in sample size and treatment protocol; as well as the inclusion of patients solely with severe periodontitis. ^25^

Although clinical studies have shown the efficacy of the one-stage full-mouth disinfection in patients with periodontitis, ^40^ this is the first to demonstrate it in obese individuals. The one-stage full-mouth disinfection protocol was chosen for this study for the following reasons: a shorter number of clinical sessions, a shorter treatment time, greater compliance, and cost-effectiveness. ^35^

*P. gingivalis, T. forsythia* , and *T.denticola,* usually called “red complex”, and *A. actinomycetemcomitans* are considered the main pathogens associated with periodontal disease. ^41^ Some speculate that obese individuals with periodontitis present higher proportions of subgingival periodontal pathogens than normal-weight individuals, increasing the risk for progressive attachment loss and periodontitis development. ^42^ In this study, obesity did not impact the changes on overall bacterial load during follow-up. Furthermore, both treatments significantly reduced *P. gingivalis* and *A. actinomycetemcomitans* at 9 months, and *T. denticola* count was only reduced within the obese group. This can be explained by the differences in *T. denticola* count at baseline. As the obese group showed a greater *T. denticola* count, it is reasonable that the periodontal treatment led to a greater reduction within this group. Periodontal therapy did not affect *T. forsythia* count in the obese and non-obese groups. Therefore, there were no major differences in microbial count when comparing obese and non-obese individuals undergoing one-stage full-mouth disinfection. These findings corroborate a recent 3-month prospective clinical study, which found no significant differences in *P. gingivalis* , *T. forsythia* , and *P. intermedia* mean counts between obese and normal-weight individuals on periodontal therapy. ^43^

Diabetes is a risk factor for periodontitis. ^44^ In this study, two individuals (5.1%) of the non-obese group and nine (16.4%) in the obese group had diabetes. Nonetheless, there was no significant difference between groups, what indicates homogeneity. Moreover, although speculated that patients with diabetes may have a worse periodontal response than non-diabetic, a recent systematic review showed that diabetes mellitus does not significantly impact periodontal clinical parameters of non-surgical periodontal therapy. ^45^ In addition, to increase this study external validity, smokers were included. There was no significant difference between groups regarding the number of smokers, but periodontal therapy is proved to be less effective within this population, ^46^ which may serve as a possible source of bias. The inclusion of overweight individuals into the control group may have presented a potential bias and a limitation for this study. Furthermore, prospective studies should assess how changes in anthropometric measurements during follow-up may impact results.

Considering the increase of obese individuals, this study results could widely contribute to dental practices. The one-stage full-mouth disinfection seemed a reliable protocol to treat periodontitis in obese individuals.

## Conclusion

Obesity did not affect the clinical and microbiological outcomes of non-surgical periodontal therapy.
